# Antiviral Ability of *Kalanchoe gracilis* Leaf Extract against Enterovirus 71 and Coxsackievirus A16

**DOI:** 10.1155/2012/503165

**Published:** 2012-05-15

**Authors:** Ching-Ying Wang, Shun-Chueh Huang, Yongjun Zhang, Zhen-Rung Lai, Szu-Hao Kung, Yuan-Shiun Chang, Cheng-Wen Lin

**Affiliations:** ^1^School of Chinese Pharmaceutical Sciences and Chinese Medicine Resources, China Medical University, Taichung 404, Taiwan; ^2^Department of Medical Laboratory Science and Biotechnology, China Medical University, Taichung 404, Taiwan; ^3^School of Pharmacy, China Medical University, Taichung 404, Taiwan; ^4^Fujian Center for Disease Control and Prevention, Fujian, Fuzhou 350001, China; ^5^Department of Nursing, Hungkuang University, Taichung 433, Taiwan; ^6^Department of Biotechnology and Laboratory Science in Medicine, National Yang Ming University, Taipei 112, Taiwan; ^7^Department of Biotechnology, Asia University, Wufeng, Taichung 413, Taiwan

## Abstract

Pandemic infection or reemergence of Enterovirus 71 (EV71) and coxsackievirus A16 (CVA16) occurs in tropical and subtropical regions, being associated with hand-foot-and-mouth disease, herpangina, aseptic meningitis, brain stem encephalitis, pulmonary edema, and paralysis. However, effective therapeutic drugs against EV71 and CVA16 are rare. *Kalanchoe gracilis* (L.) DC is used for the treatment of injuries, pain, and inflammation. This study investigated antiviral effects of *K. gracilis* leaf extract on EV71 and CVA16 replications. HPLC analysis with a C-18 reverse phase column showed fingerprint profiles of *K. gracilis* leaf extract had 15 chromatographic peaks. UV/vis absorption spectra revealed peaks 5, 12, and 15 as ferulic acid, quercetin, and kaempferol, respectively. *K. gracilis* leaf extract showed little cytotoxicity, but exhibited concentration-dependent antiviral activities including cytopathic effect, plaque, and virus yield reductions. *K. gracilis* leaf extract was shown to be more potent in antiviral activity than ferulic acid, quercetin, and kaempferol, significantly inhibiting *in vitro* replication of EV71 (IC_50_ = 35.88 *μ*g/mL) and CVA16 (IC_50_ = 42.91 *μ*g/mL). Moreover, *K. gracilis* leaf extract is a safe antienteroviral agent with the inactivation of viral 2A protease and reduction of IL-6 and RANTES expressions.

## 1. Introduction

*Kalanchoe gracilis* (L.) DC, “Da-Huan-Hun” in Chinese, is a traditional Chinese medicinal herb belonging to the *Crassulaceae* family, a folk medicine commonly used for treating injuries, pain, fever, and inflammation in Taiwan [1, 2]. Extracts of *K. gracilis* have high amounts of polyphenol and flavonoid contents that may generate potential antioxidant, anti-inflammatory, and antiproliferative activities [2, 3]. Identified constituents include coumarin, bufadienolides, flavonoids (e.g., teolin, quercetin, quercitrin, kaempferol and eupafolin), and glycosidic derivatives of eupafolin [4, 5]. The latter is a vital bioactive compound shown to possess antioxidant and anti-inflammatory effects in lipopolysaccahride-treated RAW264.7 cells as well as antiproliferating effect against HepG2 cells [3]. Coumarin-based compounds are reported with antiviral activities against Hepatitis C and human immunodeficiency virus 1 (HIV-1) [6–10]. 3-Phenyl coumarin-based compounds were previously reported to bind with viral protein R (Vpr) and inhibit Vpr-dependent HIV replication in human macrophages [10]. Chiang et al. [7] also proved less than 1 *μ*M of synthetic analogue of bis- or tetra-coumarin required to inhibit HIV-1 integrate by half. Flavonoids are reported to hold a broad spectrum of antiviral activity efficiently inhibiting replication of human rhinovirus, Sabin type 2 polio, Hepatitis A, coxsackievirus B4, and echovirus 6 [11–14]. Extract of *Kalanchoe farinacea*, another species of the *Crassulaceae* family, has shown antiviral activity against influenza A and herpes simplex virus Type 1 *in vitro *[15]. Bufadienolides identified from leaves of two other *Crassulaceae* plants, *Kalanchoe pinnata *and *K. daigremontiana x tubiflora*, inhibit activation of Epstein-Barr virus early antigen in Raji cells [16]. Together with antiviral data of related compounds and *Crassulaceae* plants, *K. gracilis* offers a valuable source of antiviral agents.

Enteroviruses (EVs) are members of the *Picornaviridae* family, which have in common a 7.4 kb single-stranded positive-sense RNA genome [17]. Enterovirus genome encodes a single large polyprotein cleaved by enteroviral 2A and 3C proteases. After protein synthesis, viral polyproteins are cleaved into four capsid (VP1 to VP4) and seven nonstructural proteins (2A, 2B, 2C, 3A, 3B, 3C, and 3D). Many crucial pathogens are enteroviruses: for example, polioviruses, coxsackievirus A (CVA) and B (CVB), echovirus EV68, EV69, EV70, EV71. CVA and EV71 are independent aetiological agents of hand-foot-and-mouth disease (HFMD), myocarditis, ocular conjunctivitis, herpangina, aseptic meningitis, or severe neurological complications, such as encephalitis and poliomyelitis-like paralysis [18]. Particularly, EV71-induced brainstem encephalitis has poor prognosis with high mortality [19]; EV71 outbreaks have been reported in countries in the Western Pacific, Sweden, and Australia [19–22]. Such epidemics in Taiwan caused 78 deaths in 1998 [23–26], 25 deaths in 2000, and 26 deaths in 2001 [21]. Importantly, cocirculations of EV71 and CVA appear in serious HFMD outbreaks in China, Taiwan, and Malaysia [26–28]. In Taiwan, coxsackievirus A16 (CVA16) is a major serotype of CVA isolates identified in HFMD outbreaks [26, 28]. Global poliovirus vaccination program plans to eradicate poliovirus diseases like severe poliomyelitis worldwide; yet no antiviral drug has been approved by FDA for treatment of EV infection. Strategy to prevent or treat EV infection proves essential, especially for EV71 and CVA16.

This study investigated *in vitro* antiviral activity of *K. gracilis* leaf extract against EV71 and CVA16, using *in vitro* models in pretreatment before virus adsorption, simultaneous treatment during infection and posttreatment after virus adsorption. All showed significantly less viral replication *in vitro*; treatment at early and late stages showed potent inhibition. This study also examined possible antiviral mechanisms: for example, inhibition of *in vitro* and cell-based 2A protease activity, anti-inflammatory effect on expression of virus-induced inflammatory genes, activation of p38 MAPK, ERK1/2, and NF-*κ*B signaling pathways.

## 2. Materials and Methods

### 2.1. Viruses and Cells

EV71 and CVA16 were obtained from clinical isolates in the Clinical Virology Laboratory, China Medical University Hospital at Taichung, Taiwan. RD cells (ATCC accession no. CCL-136) were grown in Dulbecco's Modified Eagle's Medium (DMEM; Gibco) with 10% fetal bovine serum (FBS; Gibco). All media were supplemented with 100 U/mL of penicillin and streptomycin and 2 mM of l-glutamine. HeLa-G2AwtR cells were derived from HeLa cells containing pG2AwtR plasmid that encodes an FRET probe as well as the fusion protein of red fluorescent protein (DsRed)—2Apro cleavage motif—green fluorescent protein (GFP) as described by Tsai et al. [29]. Cells were cultured in Modified Eagle's Medium (MEM; Gibco) with 10% FBS and 20 *μ*g/mL zeocin.

### 2.2. Preparation of *K. gracilis* Leaf Juice Extract

*K. gracilis* was collected from farmlands and gardens in Chiayi County, Taiwan as described in *Flora of Taiwan* and identified by Professor Hsin-Fu Yen from the National Museum of Natural Science at Taichung, Taiwan. Fresh *K. gracilis* leaves were cold-pressed, resulting leaf juice filtered through Whatman No. 1 paper, then lyophilized in a freeze dryer (IWAKI FDR-50P). This powder of leaf juice extract was stored in sterile bottles and kept in −20°C freezer.

### 2.3. Fingerprint Analysis by HPLC

For analyzing HPLC fingerprint profiles, filtered extract of *K. gracilis* leaf of ethyl acetate layer or the marker mixture of ferulic acid, quercetin, and kaempferol (10 *μ*L) was injected directly into the HPLC instrument (Shimadzu LC-10A) with a C-18 reverse phase column. Ferulic acid was purchased from Sigma-Aldrich Chemical Co (St. Louis, Mo, USA). Quercetin was purchased from Alfa Aesar-A Johnson Matthey Company. Kaempferol was purchased from Extrasynthese (France). Separation was conducted with a gradient elution of 0.25% formic acid and acetonitrile (40% between 0 and 9 min, 40–60% between 9 and 30 min) at a flow rate of 0.8 mL/min. Chromatographic peaks were detected at 260/360 nm with a 2996 PDA detector.

### 2.4. Cell Viability Assay

For cell viability, RD cells were cultured overnight on 96-well plates. Medium containing 0, 1, 50, 250, 500, or 1000 *μ*g/mL of *K. gracilis* leaf extract was added and incubated for 24 and 48 hours, followed by MTT assay. Survival rates of cells were calculated as the ratio of optical density at 570–630 nm (OD_570–630_) of treated cells to OD_570–630_ of untreated cells. Quintuplicate wells were analyzed for each concentration. The data represented the means ± SD of three independent experiments. Cytotoxic concentration of 50% toxic effect (CC_50_) was determined using a computer program (provided by John Spouge, National Center for Biotechnology Information, National Institutes of Health). Ferulic acid, quercetin, and kaempferol at concentrations of 1, 10, 50, 100, and 500 *μ*g/mL were also tested for cytotoxicity.

### 2.5. Cytopathic Effect (CPE) Reduction Assay

RD cells (4 × 10^5^ RD cells per well) were seeded to the 6-well plate. Next day, EV71 or CVA16 at the multiplicity of infection (MOI) of 1 was mixed with Dulbecco's modified Eagle's medium containing 2% FBS alone or medium with 100 *μ*g/mL of *K. gracilis* leaf extract before adding to the RD cells to incubate for 24 and 48 h at 37°C. Cell morphology was observed and photographed under microscopy.

### 2.6. Plaque Reduction Assay

RD cells (4 × 10^5^ RD cells per well) were seeded to the 6-well plates. Next day, cells were infected with EV71 or CVA16 (100 pfu) in the absence or presence of *K. gracilis* leaf extract at 10, 50, or 100 *μ*g/mL or the marker compound (quercetin, ferulic acid, and kaempferol) at 0.1, 1, 10, and 50 *μ*g/mL 1 h at 37°C. Then, 2 mL of medium containing 3% agarose was overlaid onto the cells and incubated for 2 days at 37°C in a CO_2_ incubator. At the end of incubation, cells were stained with 0.1% Crystal Violet. Ferulic acid, quercetin, and kaempferol at the concentrations of 0.1, 1, 10, and 50 *μ*g/mL were also tested for plaque reduction. The concentrations that reduced the number of plaques by 50% (IC_50_) were then determined. The therapeutic index (TI) was determined by the ratio of CC_50_ to IC_50_.

### 2.7. Virucidal Activity Assay

EV71 or CVA16 (10^4^ pfu) was mixed with test compounds of various concentrations (0, 50, and 100 *μ*g/mL) or medium and incubated for 30 min at room temperature, then serial dilutions of the mixture were performed using the plaque assay. The residual infectivity was measured as described in the plaque assay.

### 2.8. Virus Yield and Time-of-Addition Assays

*K. gracilis* leaf extract was added to RD cells before (30 min pretreatment), during (simultaneous treatment), and after (30 min posttreatment) the viral infections of EV71 and CVA16 at the titer of 300 pfu. 48 h after infection, culture media were collected and RNA genomes were extracted by QIAamp Viral RNA Mini Kit (Qiagen). Real-time RT-PCR was performed with specific primers, SYBR green PCR Master Mix, and SYBR green I dsDNA binding dye. Oligonucleotides for VP1 were forward primer 5′-GGCCCCTGAATGCGGCTAATCC-3′  (nt 458–480) and reverse primer 5′-GCGATTGTCA CCATWAGCAGYCA-3′ (nt 603–581) as described in the report of Oberste et al. [30]. PCR product level was monitored with an ABI PRISM 7000 sequence detection system (Applied Biosystems). For comparing the viral RNA load in the cultured media, a delta Ct value was calculated by subtracting the Ct value for viral load in cultured media of *K. gracilis *leaf-extract-treated infected cells from the Ct value for viral load in cultured media of infected cells without treatment. If the delta Ct value was greater than 3.3, indicating 50 *μ*g/mL of *K. gracilis* leaf extract had more than 1-log reduction (equal to 90% effective concentration [EC_90_]) in virus RNA loads.

### 2.9. Fluorescence Resonance Energy Transfer (FRET) Assay

HeLa-G2AwtR cells (1 × 10^6^ cells per well) expressing the FRET probe were seeded to the 6-well tissue culture plates and infected with EV71 or CVA16 (1 × 10^6^ pfu). After 1 h of adsorption, the inoculum was aspirated and 200 *μ*L of modified Eagle's medium containing 2% FBS alone or medium with 1, 10, 50, 100, or 150 *μ*g/mL of *K. gracilis* leaf extract was added to each well. Cells were harvested 48 h after infection, and lysates were transferred to 96-well plates for fluorescence. The fluorescent intensity of the FRET probes was measured by a fluorescent-plate reader with an excitation wavelength at 390/20 nm (for GFP^2^ at 510/10 nm) and an emission wavelength at 590/14 nm (for DsRed2), in which DsRed2 was excited by the emission of GFP^2^ at 510/10 nm. The data presented are mean values of three independent experiments, and the error bars represent standard deviations.

### 2.10. Construction, Expression, and Purification of EV71 2A Protease

The gene encoding EV71 2A protease was amplified using PCR with cDNA of EV71 isolate CMUH01. The forward primer 5′-GCGCGGATCCGGGAAATTCGGTCAGCAG-3′ and the reverse primer 5′-GCGCCTCGAGCTGCTCCATCGCTTCCTC-3′, which contain BamHI and XhoI restriction enzyme sites (underlined), were used for C-terminal His-tagged protease. PCR products were digested with BamHI and XhoI, and the DNA fragment was cloned into pET24a (Novagen). The plasmid containing the protease gene was subsequently transformed into *E. coli* Origami B (DE3) (Novagen) for protein expression. A 10 mL overnight culture of a single colony was used to inoculate 400 mL of fresh LB medium containing 25 *μ*g/mL kanamycin. Cells were grown to an A600 of 0.6 and induced with 1 mM IPTG. After IPTG induction overnight, cells were harvested by centrifugation at 6000 rpm for 30 min, and then resuspended in a denaturing buffer (10 mM imidazole, 8 M urea and 1 mM *β*-mercaptoethanol) before subjecting to sonication. Separation of the supernatant was conducted in Ni-NTA column, and His-tagged 2A protease was allowed to renature slowly by gradient washing with refolding buffer (10 mM imidazole and 1 mM *β*-mercaptoethanol) overnight. Finally, recombinant 2A protease was eluted with 25 mM Tris-HCl, pH 7.5, 150 mM NaCl, and 300 mM imidazole. The concentration of purified protein was determined using Bio-Rad protein assay.

### 2.11. *In Vitro* 2A Protease Activity Assay

In the protease activity assay, 2A protease was designed to interact with horseradish peroxidase (HRP). HRP is a commercially available substrate, and its Leu-Gly pairs at residues 122-123 correspond to the cleavage site by 2A protease. To examine the most suitable concentration of 2A protease for interaction with HRP, 2A proteases of various concentrations ranging from 1 *μ*g/mL to 150 *μ*g/mL were each added and incubated with 1 *μ*g/mL of HRP for 2 h at 37°C. Mixtures were then developed with ABTS/H_2_O_2_ and measured at OD_405_. The extract of *K. gracilis* leaf was tested for anti-EV71 2A protease activity, thus it was incubated with HRP for 2 h at 37°C in 96-well plates *in vitro*. The remaining activity of HRP in each reaction was determined with a chromogenic substrate ABTS/H_2_O_2_ and the intensity of the developed color was measured at 405 nm. Percentage of inhibition of EV71 2A protease activity was calculated by the following equation: (OD405_HRP+drug+2Apro_ − OD405_HRP+2Apro_)/(OD405_HRP  only_ − OD405_HRP+2Apro_) × 100%.

### 2.12. Quantification of Proinflammatory Gene Expression Using Real-Time RT-PCR

Total RNA was isolated from RD cells with or without the treatment of *K. gracilis* leaf extract and with or without virus infection via purification kit (PureLink Micro-to-Midi total RNA Purification System, Invitrogen). cDNA was synthesized from 1000 ng total RNA with oligo dT primer and SuperScript III reverse transcriptase kit (Invitrogen). To gauge expression in response to treatment of *K. gracilis* leaf extract and/or virus infection, a two-step RT-PCR using SYBR Green I was used. The following oligonucleotide primer pairs were used in the present study for the detection of cytokines, including (1) forward primer 5′-TCCCCATATTCCTCGGAC-3′ and reverse primer 5′-GATGTACTCCCGAACCCA-3′ for human RANTES, (2) forward primer 5′-ATGCCCCAAGCTGAGAACCAAGACCCA-3′ and reverse primer 5′-TCTCAAGGGGCTGGGTCAGCTATCCCA-3′ for human IL-10, (3) forward primer 5′-GATGGATGCTTCCAATCTGGAT-3′ and reverse primer 5′-AGTTCTCCATAGAGAACAACATA-3′ for human IL-6, (4) forward primer 5′-CTCTAGAGCACCATGCTACAGAC-3′ and reverse primer 5′-TGGAATCCAGGGGAAACACTG-3′ for human IL-1*α*, and (5) forward primer 5′- CATGCGTTTCCGTTACAAGTGCGA-3′ and reverse primer 5′-TGGGTGCGTCTTAGTGGTATCTGT-3′ for human NF-*κ*B. In addition, glyceraldehyde-3-phosphate dehydrogenase (GAPDH) mRNA, a housekeeping gene, was measured using 5′-AGCCACATCGCTCAGACAC-3′ and 5′-GCCCCA ATACGACCAAATCC-3′ as forward and reverse primers. Real-time PCR reaction mixture contained 2.5 *μ*L of cDNA (reverse transcription mixture), 200 nM of each primer in SYBR Green I master mix (LightCycler TaqMAn Master, Roche Diagnostics). PCR was performed by an amplification protocol consisting of 1 cycle at 50°C for 2 min, 1 cycle at 95°C for 10 min, 40 cycles at 95°C for 15 sec, and 60°C for 1 min. Specific products were amplified and detected in ABI PRISM 7700 sequence detection system (PE Applied Biosystems), and relative changes in mRNA levels of indicated genes were gene normalized by housekeeping gene GAPDH.

### 2.13. Western Blot Analysis

RD cells were infected with the virus (8 × 10^5^ pfu) in the presence or absence of *K. gracilis* leaf extract. The lysate proteins of RD cells were dissolved in 2x SDS-PAGE sample buffer without 2-mercaptoethanol, boiled for 10 min, and then separated on 8% SDS-PAGE gels before being transferred to nitrocellulose paper. Resultant blots were blocked with 5% skim milk then reacted with properly diluted monoclonal antibodies including anti-ERK1/2, anti-phospho-ERK1/2, anti-p38 MAPK, anti-phospho-p38 MAPK, anti-p65, anti-phospho-p65, anti-caspase 9 (Cell Signaling Technology) and anti-*β*-actin. The immune complexes were detected using horseradish peroxidase-conjugated goat anti-mouse IgG antibodies followed by enhanced chemiluminescence reaction (Amersham Pharmacia Biotech).

### 2.14. *In Vivo* Anti-EV71 Assay

Suckling mice of 1-day-old were intraperitoneally inoculated with EV71 (1.7 × 10^5^ pfu) and intraperitoneally injected with *K. gracilis *leaf extract (0, 1, 2 mg/kg) once on day 1, 3, 5, 7 after-infection. 3 mice from each group were sacrificed on day 2, 4, 6, and 8. Intestine samples were collected for detection of virus loads using real-time RT PCR.

### 2.15. Statistical Analysis

ANOVA analysis using SPSS program (version 10.1, SPSS Inc., IL, USA) or Scheffe test was used to analyze all data. *P* value less than 0.05 was considered statistically significant.

## 3. Results

### 3.1. Fingerprint Profiling of *K. gracilis* Leaf Extract

**Figure 1:**
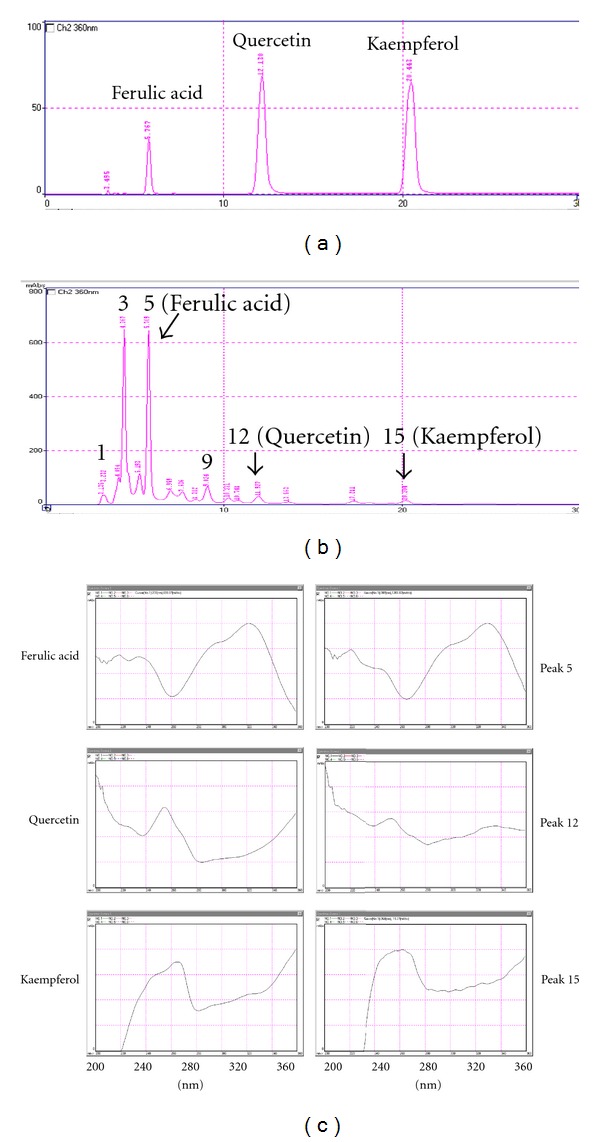
HPLC fingerprint profiles and UV/vis absorption spectra of *K. gracilis *leaf extract of ethyl acetate layer and marker components. Marker components of ferulic acid, quercetin, and kaempferol (a) and *K. gracilis *leaf extract of ethyl acetate layer (b) were analyzed using HPLC with a C-18 reverse phase column. Eluents were detected at 360 nm. Maximum absorption wavelengths of ferulic acid, quercetin, kaempferol, and chromatographic peaks 5, 12, and 15 were measured by UV/vis absorption spectra (200–360 nm).

To determine the fingerprint of *K. gracilis *leaf extract, the ethyl acetate layer of filtered leaf extract and marker components of ferulic acid, quercetin, and kaempferol as well were analyzed using HPLC with a C-18 reverse phase column (Figure 1). The retention time was found at 5.74 min for ferulic acid, 12.13 min for quercetin, and 20.44 min for kaempferol at 360 nm (Figure 1(a)). HPLC chromatographic peaks of *K. gracilis *leaf extract of ethyl acetate layer indicated the retention times of peaks 5, 12, and 15 were identical to those of ferulic acid, quercetin, and kaempferol, repectively (Figure 1(b)). UV absorption spectra (200–360 nm) of peaks 5, 12, and 15 were identical as compared to the marker components of ferulic acid, quercetin, and kaempferol, respectively (Figure 1(c)). HPLC and UV absorption analyses revealed fingerprint profiling as well as the maker components in *K. gracilis *leaf extract.

### 3.2. Cytotoxicity of *K. gracilis* Leaf Extract in RD Cells

**Figure 2:**
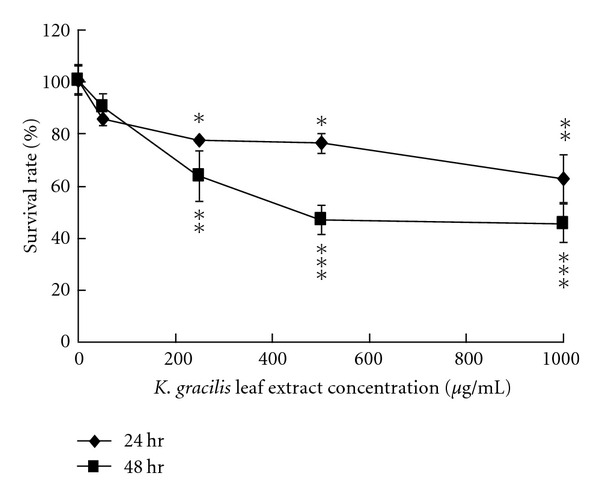
Cytotoxicity of* K. gracilis *leaf extract. RD cells were cultured overnight on 96-well plates. Serial dilutions of *K. gracilis* leaf extract were added and incubated for 24 and 48 hours, followed by MTT assay. Survival rates of cells were calculated as the ratio of optical density at 570–630 nm (OD_570–630_) of treated cells to OD_570–630_ of untreated cells. *P value < 0.05; **P value < 0.01; ***P value < 0.001 by Scheffe's test.

To evaluate the cytotoxicity of *K. gracilis *leaf extract, RD cells were treated with *K. gracilis* leaf extract at the concentration range of 1–1000 *μ*g/mL.* In vitro* cytotoxicity assay showed that *K. gracilis *leaf extract was not cytotoxic to RD cells in the concentration range of 1–500 *μ*g/mL 24 and 48 h posttreatment (Figure 2). 50% cytotoxicity concentrations (CC_50_) of ferulic acid, quercetin, and kaempferol were all greater than 500 *μ*g/mL, and so was that of *K. gracilis *leaf extract (data not shown). The results also demonstrated *K. gracilis *leaf extract as being less cytotoxic in comparison with the 3 marker components.

### 3.3. Inhibitory Effect of *K. gracilis* Leaf Extract on the Replication of EV71 and CVA16

**Figure 3:**
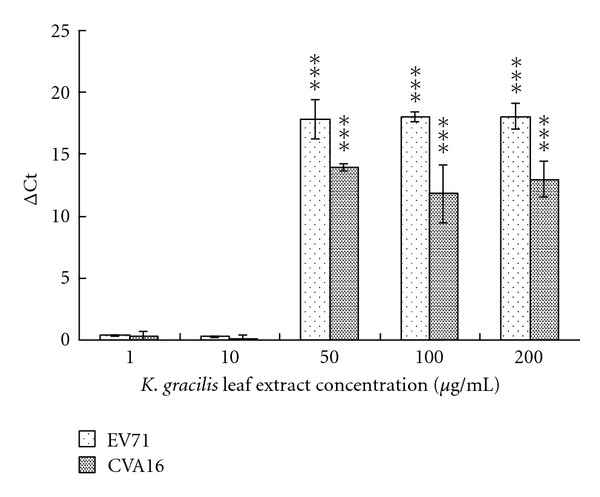
Reduction of virus yields by *K. gracilis *leaf extract. Each virus at the titer of 300 pfu was mixed with *K. gracilis *leaf extract and then added into RD cell cultures. Virus titers in each cultured supernatant were measured 48 h after infection using real-time RT-PCR. The delta Ct value was calculated by subtracting the Ct value for viral load in cultured media of *K. gracilis *leaf-extract-treated infected cells from the Ct value for viral load in cultured media of infected cells without treatment. Assay was performed as described in the Section 2. *P value < 0.05; **P value < 0.01; ***P value < 0.001 by Scheffe test.

**Figure 4:**
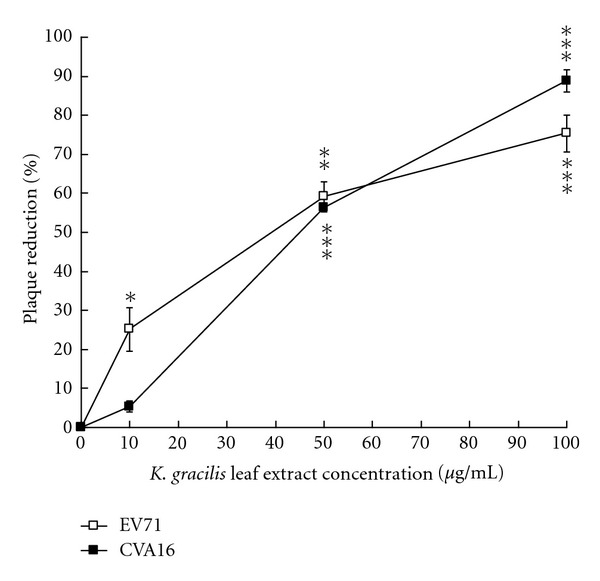
Plaque reduction of *K. gracilis* leaf extract. RD cells were infected with EV71 or CVA16 at a titer of 100 pfu and simultaneously treated with* K. gracilis* leaf extract at concentrations of 0, 10, 50, and 100 *μ*g/mL. After 1 h incubation, RD cells were covered with agarose overlay medium and incubated for 2 days at 37°C in a CO_2_ incubator. Finally, the cells were stained with 0.1% crystal violet. *P value < 0.05; **P value < 0.01; ***P value < 0.001 by Scheffe test.

**Figure 5:**
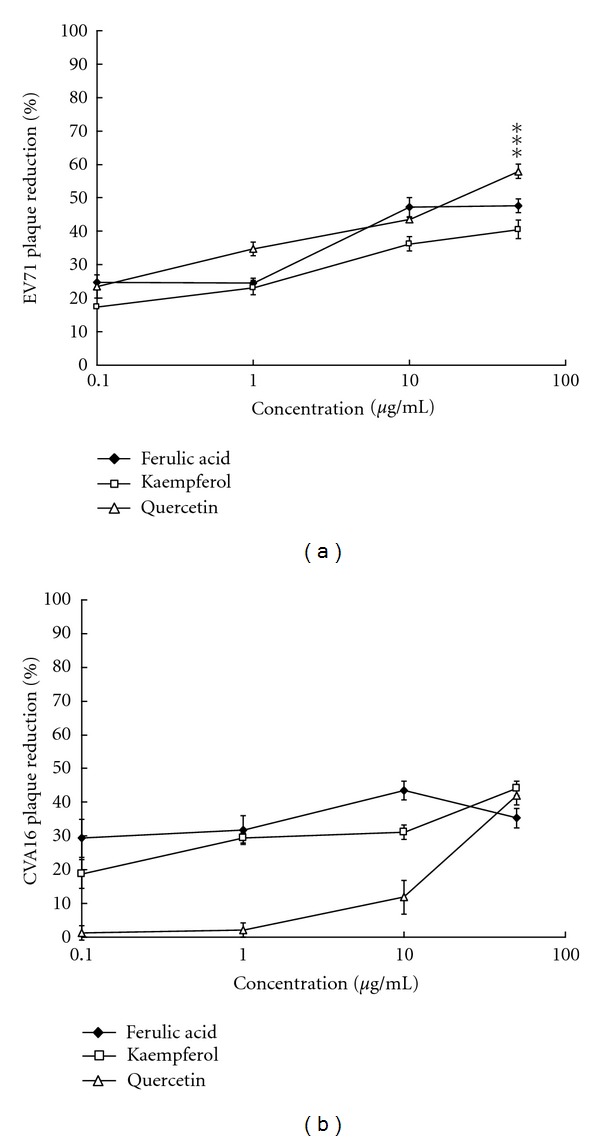
Plaque reduction of quercetin, ferulic acid and kaempferol. RD cells were infected with EV71 (a) or CVA16 (b) at a titer of 100 pfu and simultaneously treated with quercetin, ferulic acid, and kaempferol at concentrations of 0, 0.1, 1, 10, and 50 *μ*g/mL. After 1 h incubation, RD cells were covered with agarose overlay medium and incubated for 2 days at 37°C in a CO_2_ incubator. Finally, the cells were stained with 0.1% crystal violet. *P value < 0.05; **P value < 0.01; ***P value < 0.001 by Scheffe test.

The anti-viral effects of *K. gracilis *leaf extract against EV71 and CVA16 were further evaluated in virucidal activity, cytopathic effect (CPE) reduction, viral plaque reduction, and virus yield reduction assays (see supplemental Figure 1 in Supplementary Material available online at doi:10.1155/2012/503165, Figures 3 and 4). *In vitro* incubation with 100 *μ*g/mL of *K. gracilis *leaf extract for 30 min at room temperature had no significant effect on EV71 or CVA16 infectivity, indicating *K. gracilis *leaf extract had no virucidal activity against EV71 and CVA16 (data not shown). However, when *K. gracilis* leaf extract was added to EV71- and CVA16-infected RD cells, significant inhibition on EV71- and CVA16-induced CPE was observed, as shown in Supplemental Figures 1(a) and 1(b). For detection of virus yields in RD cells, the culture supernatants of EV71- and CVA16-infected RD cells with and without simultaneous-treatment of *K. gracilis *leaf extract in the concentration range of 1–200 *μ*g/mL were harvested on day 2 and analyzed for viral RNA loads, using quantitative real-time RT-PCR (Figure 3). Real-time RT-PCR assay indicated that viral RNA loads in the supernatant of infected cells treated with 50 *μ*g/mL of* K. gracilis *leaf extract (a Ct value of 31.68 for EV71 and a Ct value of 28.20 for CVA16) were significantly lower than those of infected cells without* K. gracilis *leaf extract treatment (a Ct value of 13.82 for EV71 and a Ct value of 14.25 for CVA16) (Figure 3). Moreover, treatment with* K. gracilis *leaf extract at 50 *μ*g/mL had more than 1-log reduction in virus RNA loads since its delta Ct value was greater than 3.3. In addition, *K. gracilis *leaf extract exhibited concentration-dependent inhibitory effects on the replication of EV71 and CVA16 using plaque assay (Figure 4). IC_50_ value of *K. gracilis *leaf extract in plaque reduction assay was 35.88 *μ*g/mL for EV71 and 42.91 *μ*g/mL for CVA16. Moreover, quercetin, but not ferulic acid and kaempferol, showed a concentration-dependent manner on plaque reduction (IC_50_ = 39.63 *μ*g/mL for EV71; IC_50_ = 59.53 *μ*g/mL for CVA16) (Figure 5). The therapeutic index (TI) of *K. gracilis *leaf extract was greater than 10 against EV71 and CVA16, while quercetin TI was less than 10 against CVA16. *K. gracilis *leaf extract demonstrated higher antiviral potential than its marker component quercetin in treatment of EV71 and CVA16 infections.

### 3.4. Antiviral Effect in Different Stages of EV71 and CVA16 Infections

**Figure 6:**
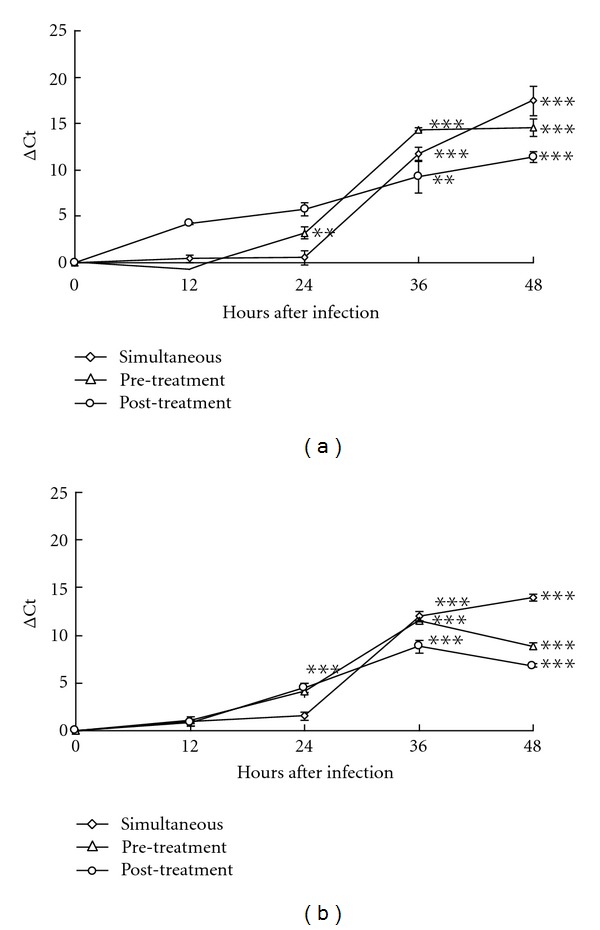
Time of addition assay for analysis of inhibitory effects of* K. gracilis* leaf extract on viral replication processes. *K. gracilis* leaf extracts at concentrations of 0 and 50 *μ*g/mL were added to RD cells before (pre-treatment), during (simultaneous treatment), and after (post-treatment) infection with EV71 (a) or CVA16 (b) at the titer of 300 pfu. Virus titer in each cultured supernatant was measured 48 h after infection using real-time RT-PCR. Assay was performed as described in Section 2. *P value < 0.05; **P value < 0.01; ***P value < 0.001 by Scheffe's test.

To examine the possible mechanisms of *K. gracilis *leaf extract against EV71 and CVA16, the antiviral activity of *K. gracilis *leaf extract was further determined using time of addition studies (Figure 6). RD cells were treated with 50 *μ*g/mL of *K. gracilis *leaf extract 30 min before, during, and 30 min after infection. Viral RNA quantitative real-time PCR demonstrated that* K. gracilis *leaf extract reached 90% yield reduction of EV71 at 12 h by the post-treatment procedure and at 36 h by the pre-treatment and simultaneous treatment procedures (Figure 6(a)). In addition, pre- and post-treatments of *K. gracilis *leaf extract both exerted 90% reductions in the yield of CVA16 at 24 h, and simultaneous-treatment of *K. gracilis *leaf extract achieved 90% reduction at 36 h (Figure 6(b)). The results showed antiviral effects of *K. gracilis *leaf extract on EV71 and CVA16 replications, being possibly associated with targeting viral protein function, reducing virus-induced cytopathogenesis, or inducing host antiviral responses.

### 3.5. Inhibition of Viral 2A Protease Activity in Cell-Based FRET by *K. gracilis* Leaf Extract

Since enterovirus 2A protease that cleaves viral polyproteins and cellular factors has multifaceted activities for regulating different viral and cellular processes including viral replication and cytopathogenesis, particular in apoptosis and innate immunity [31], the possible inhibitory mechanisms of *K. gracilis* leaf extract on viral 2A protease was tested using a cell-based FRET assay of viral 2A protease activity. In general, a stable cell line continuously expressing GFP2-DsRed2 fusion protein connected by 2A protease cleavage site was used (HeLa-G2AwtR cells; GRTTLSTR↓GPPRGGPG; ↓ indicates cleavage site). The fusion protein would be cleaved by viral 2A protease at time of infection. To test FRET efficiency, HeLa-G2AwtR cells were infected with EV71 or CVA16 at MOIs of 0.25, 0.5, and 1 for 48 h and were harvested and subjected to measurement by a fluorescent-plate reader. The FRET ratio was defined as the intensity of emission at 590/14 nm for DsRed2 divided by that of GFP^2^ at 510/10 nm. Compared with the mock infection control, infected HeLa-G2AwtR cells exhibited a decline in percentage of the emission intensity of FRET probes in a virus titer-dependent manner (data not shown).

**Figure 7:**
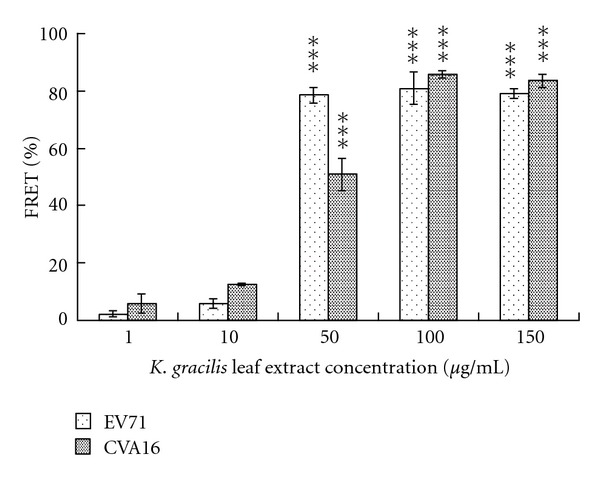
Cell-based FRET assays for characterization of inhibitory effects of* K. gracilis* leaf extract on virus 2A protease enzymatic activity. HeLa-G2AwtR cells were infected with EV71 or CVA16 at a MOI of 1, and the cells were washed with MEM and then treated with *K. gracilis* leaf extract at the dosages of 1, 10, 50, 100, and 150 *μ*g/mL. 12, 24, 36, and 48 h after infection, cells were harvested and subjected to measurement by a fluorescent-plate reader with the excitation wavelength at 390/20 nm and emission wavelength at 510/10 nm (for GFP^2^) or 590/14 nm (for DsRed2). FRET ratio was defined as intensity of emission at 590/14 nm divided by that at 510/10 nm. *P value < 0.05; **P value < 0.01; ***P value < 0.001 by Scheffe test.

To further evaluate the inhibitory effects of *K. gracilis *leaf extract on the emission intensity of FRET probes in infected cells, HeLa-G2AwtR cells were infected with EV71 or CVA16 at MOI = 1 for 48 h. After 60 min of adsorption, the inoculum was aspirated and 200 *μ*L of medium (modified Eagle's medium containing 2% FBS) alone or medium with *K. gracilis *leaf extract at various concentrations was added to each well (Figure 7). As shown in Figure 7, *K. gracilis *leaf extract increased the emission intensity of FRET probes in cells infected with EV71 and CVA16, exhibiting inhibitory effect on enteroviral 2A protease activity in a dose-dependent manner. IC_50_ values of *K. gracilis *leaf extract in cell-based FRET assay were 40.82 *μ*g/mL for EV71 and 47.84 *μ*g/mL for CVA16. The result implied *K. gracilis *leaf extract inhibited the proteolytic activity of EV71 and CVA16 2A proteases.

### 3.6. Inhibition of *In Vitro* 2A Protease Activity by *K. gracilis* Leaf Extract

**Figure 8:**
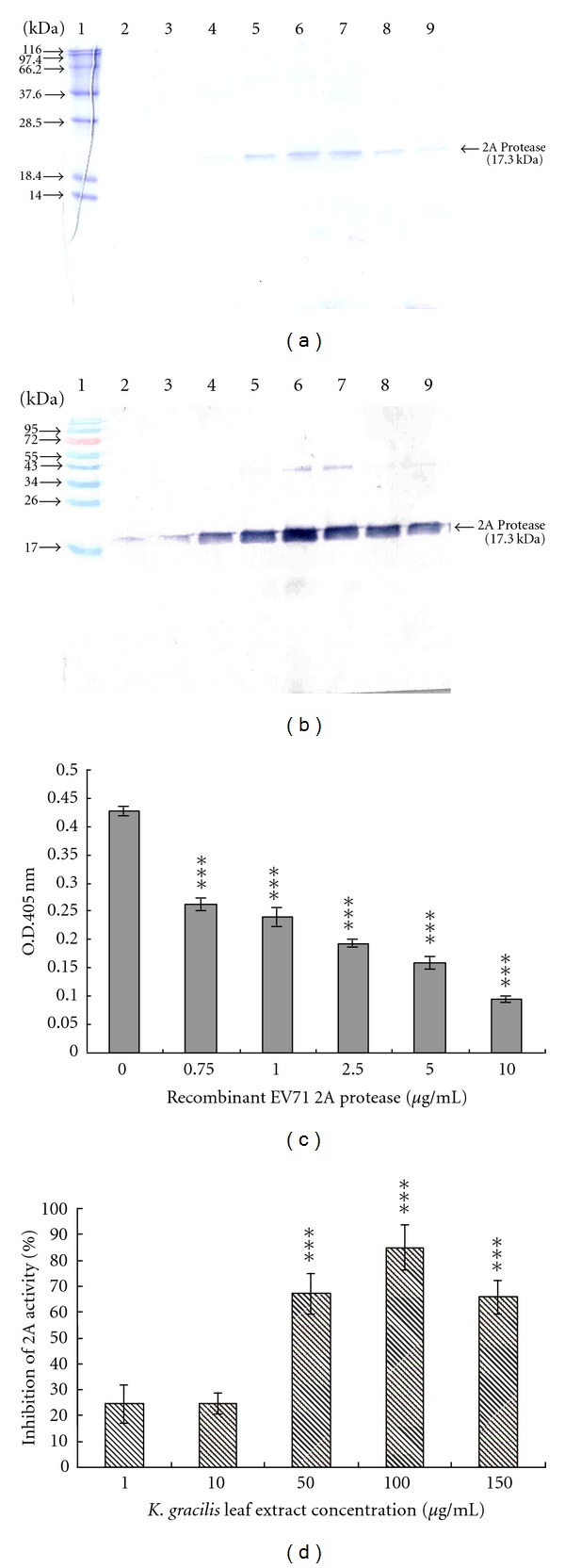
*In vitro* recombinant 2A protease enzymatic assay. SDS-PAGE (a) and Western blot analysis (b) of the purified 2A protease. Recombinant 2A protease protein was expressed after induction of IPTG treatment and purified through fusion His-tag bound to Ni-NTA agarose column. Purified recombinant 2A protease proteins were resolved on 10% SDS-PAGE and transferred onto nitrocellulose paper. The blot was probed with anti-His-tag and developed with an alkaline phosphatase-conjugated secondary antibody and NBT/BCIP substrates. Lane 1 represents protein molecular weight markers. Lanes 2–9 show the fractions of the purified 2A protease proteins. For characterization of recombinant 2A protease activity *in vitro* (c), purified 2A protease at concentrations of 0, 0.75, 1, 2.5, 5 and 10 *μ*g/mL were incubated with HRP (0.5 *μ*g/mL concentration) for 2 h at 37°C. Mixtures were developed with ABTS/H_2_O_2_ and measured at OD_405_. For inhibitory enzymatic assays (d), *K. gracilis* leaf extract at concentration of 1, 10, 50, 100, or 150 *μ*g/mL was added into the mixture of purified 2A protease and HRP then incubated for 2 h at 37°C in 96-well plates *in vitro*. Mixtures were developed with ABTS/H_2_O_2_ and measured at OD_405_. Percentage of inhibition of 2A protease activity was calculated as (OD405_HRP + drug + 2A protease_ − OD405_HRP + 2A protease_)/(OD405_HRP  only_ − OD405_HRP + 2A protease_) ×  100%. *P value < 0.05; **P value < 0.01; ***P value < 0.001 by Scheffe's test.

To confirm the inhibitory effect of *K. gracilis *leaf extract on viral 2A protease, *in vitro* cleavage assay of recombinant 2A protease was performed using horseradish peroxidase-based substrate. The cDNA fragment of EV71 2A protease gene was cloned into pET24a expression vector and in-frame-fused with a C-terminal hexa-His-tag. EV71 2A protease protein was expressed in the recombinant *E. coli* cells after IPTG induction and purified using immobilized-metal affinity chromatography (IMAC) (Figure 8(a)). Western blot analysis of purified recombinant 2A protein with anti-His-tag antibody revealed an immunoreactive band near 17.3 kDa, matching closely to the expected molecular weight (Figure 8(b)). Recombinant 2A proteases of various concentrations (0.75, 1, 2.5, 5, and 10 *μ*g/mL) were each added and incubated with HRP. The uncleaved HRP in each reaction was then incubated with ABTS/H_2_O_2_ and the remaining HRP activity measured at OD_405_. As shown, OD_405_ exhibited a reverse dose-dependent relationship with the increase of 2A protease (Figure 8(c)). IC_50_ value of *K. gracilis *leaf extract in 2A protease assay was 32.46 *μ*g/mL for EV71. Moreover, this *in vitro* cleavage assay of recombinant 2A protease using HRP-based enzymatic method demonstrated *K. gracilis *leaf extract processed a specific inhibition of 2A protease.

### 3.7. Inhibition of Virus-Induced Apoptosis and Proinflammatory Gene Expression by *K. gracilis* Leaf Extract

**Figure 9:**
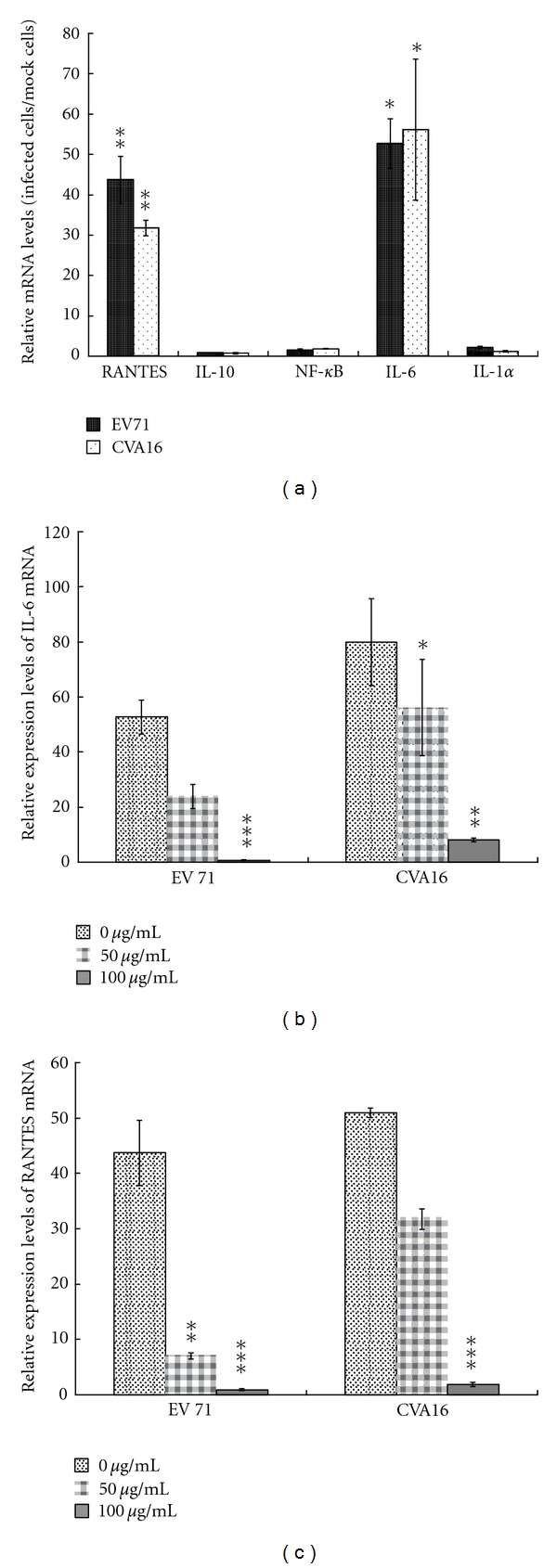
Relative levels of pro-inflammatory gene expression in infected RD cells with or without the treatment of *K. gracilis* leaf extract. For detection of virus-induced proinflammatory gene expression (a), RD cells were infected with EV71 or CVA16 for 8 h then harvested for RNA extraction. Real-time RT-PCR was performed as described in Section 2. For detection of inhibitory effect of *K. gracilis* leaf extract on virus-induced proinflammatory gene expression, EV71- or CVA16-infected cells (b, c) were simultaneously treated with* K. gracilis* leaf extract at concentrations of 0, 50, and 100 *μ*g/mL. After 8 h of incubation, total RNA was isolated from each group and Real-time RT-PCR was performed as described in Section 2. *P value < 0.05; **P value < 0.01; ***P value < 0.001 by Scheffe's test.

Since 2A protease was associated with EV71-induced apoptosis [32], Western blotting analysis of EV71-infected cells with or without the treatment of K. gracilis leaf extract was performed using anti-caspase 9 antibodies (Supplemental Figure 3). K. gracilis leaf extract significantly reduced the proform and active form of caspase 9 in EV71-infected cells, indicating that the inhibitory effect of K. gracilis leaf extract on 2A protease correlated with the decrease of EV71-induced apoptosis. Moreover, 2A protease interferes nuclear-cytoplasmic trafficking of NF-*κ*B via the cleavage of FG nucleoporins [31]; the present study further examined whether *K. gracilis *leaf extract inhibited virus-induced inflammatory gene expression and induced signal pathways by quantitative real-time PCR and Western blot assay (Figure 9 and Supplemental Figure 2). Both EV71 and CVA16 caused significant increases in IL-6 and RANTES mRNA levels; on the other hand, IL-10, NF-*κ*B p65 and IL-1*α* were not elevated in RD cells 8 h after infection (Figure 9(a)). Treatment of* K. gracilis *leaf extract showed concentration-dependent inhibitions of EV71- and CVA16-induced IL-6 and RANTES expressions (Figures 9(b) and 9(c)), indicating significant inhibitory effect on EV71- and CVA16-induced inflammations. Subsequently, phosphorylation of p38 MAPK, ERK1/2, and NF-*κ*B p65 in virus-infected RD cells with or without treatment of* K. gracilis *leaf extract was analyzed by Western blotting with phosphorylation site-specific antibodies (Supplemental Figure 2). EV71 and CVA16 infections caused time-dependent increases of p38 MAPK, ERK1/2, and NF-*κ*B p65 phosphorylation. Treatment with *K. gracilis *leaf extract in infected cells inhibited NF-*κ*B p65 phosphorylation 4 h after infection. Results demonstrated that inhibition of EV71- and CVA16-induced IL-6 and RANTES expressions correlated with the reduction of NF-*κ*B p65 activation in RD cells and suggested that the antiapoptotic and anti-inflammatory effect of *K. gracilis *leaf extract was involved in its antiviral activity against EV71 and CVA16.

### 3.8. *In Vivo* Antiviral Activity by *K. gracilis* Leaf Extract

To evaluate *in vivo* antiviral activities of* K. gracilis *leaf extract, viral loads were examined in intestine samples from EV71-inoculated suckling mice with or without treatment of *K. gracilis *leaf extract (Supplemental Table 1). In a suckling mouse model, EV71 were detectable in intestine samples on days 2, 4, and 6 after intraperitoneal inoculation. Effect of treatment with *K. gracilis *leaf extract in EV71 EV71-inoculated suckling mice demonstrated an undetectable virus load in intestine samples on day 2 by one intraperitoneal injection of 2 mg/kg of* K. gracilis *leaf extract and on day 6 by three intraperitoneal injections of 1 mg/kg of* K. gracilis *leaf extract. Results revealed *in vivo* antiviral activity of *K. gracilis *leaf extract as well as a concentration-dependent manner on inhibiting EV71 replication in the intestine of suckling mice.

## 4. Discussion

*K. gracilis* is a traditional Chinese medicinal herb commonly used for the treatments of injury, pain, and inflammation; such effects have also been evidenced in animal experiments of formalin-induced nociception and *λ*-carrageenan-induced inflammation recently [2]. This study demonstrated *K. gracilis* leaf extract exhibiting the antiviral activity against EV71 and CVA16 *in vitro* and *in vivo* (Figures 3–8, Supplemental Table 1). The results indicated *K. gracilis *exhibiting antiviral effects similar to other species in the Kalanchoe genus. For example, *K. pinnata* Pers. has been shown with significant antiviral activity against Herpes Simplex and Epstein-Barr viruses [16]. *K. farinacea* inhibits *in vitro* replication of Influenza A and Herpes Simplex Virus Type 1 [15]. *K. brasiliensis* possesses anti-inflammatory and immunosuppressive effects on zymosan-induced inflammation in mice [33]. Extracts of *Kalanchoe farinacea* have *in vitro* antiviral activity against Influenza A and Herpes Simplex Type 1 [15]. Together with the results and the previous reports, herb species in the Kalanchoe genus could possess active antiviral compounds.

HPLC and UV/vis absorption spectra demonstrated the chemical contents and the fingerprint profile of *K. gracilis* leaf extract of ethyl acetate layer by using a photodiode array detector (Figure 1). The relative amounts of ferulic acid (~7.59% w/w), quercetin (~0.33% w/w), and kaempferol (~0.16% w/w) were also identified in *K. gracilis* leaf extract of ethyl acetate layer and were found to be in agreement with identified flavonoids of *K. gracilis* in previous reports [3–5]. Flavonoids exhibit multiple functions, of which inhibition on viral replication [11–14] is among. In the present study, quercetin was demonstrated to possess concentration-dependent antienteroviral activities with the IC_50_ values of 39.63 *μ*g/mL for EV71 and 59.53 *μ*g/mL for CVA16 (Figure 5). However, anti-enteroviral activity of quercetin was less effective than that of *K. gracilis* leaf extract (Figure 4). *K. gracilis* leaf extract is suggested to contain other potent anti-enteroviral components besides quercetin. Phytochemically, *Kalanchoe* species are known to contain flavonoids (e.g., teolin, quercetin, quercitrin, kaempferol, eupafolin), glycosidic derivatives of eupafolin, bufadienolides, and coumarins [4, 5, 16], of which bis- and tetra-coumarins have been shown with inhibitory effects on the activity of HIV-1 integrase [7], while bufadienolides can reduce the activation of Epstein-Barr virus early antigen [16]. In addition, many active compounds of natural products have been identified as potential anti-EV71 agents, including gallic acid (IC_50_ = 0.8 *μ*g/mL) [34], allophycocyanin (IC_50_ = 0.056 *μ*M) [35], chrysosplenetin (IC_50_ = 0.2 *μ*M), penduletin (IC_50_ = 0.2 *μ*M) [36], and aloe-emodin (IC_50_ = 0.14 *μ*M) [37]. This study revealed that quercetin showed a specific anti-EV71 activity (IC_50_ = 39.6 *μ*g/mL), but might be less potent than the active compounds of natural products in above previous reports. Therefore, active phytochemical contents of K. gracilis leaf extract against EV71 and CVA16 should be further characterized.

In the evaluation of cytotoxicity of *K. gracilis *leaf extract, it was shown that it manifested less cytotoxicity in comparison with ferulic acid, quercetin, and kaempferol. Not only that, it showed better antiviral activities against EV71 and CVA16 as compared to the three marker components (Figures 2–4, Supplemental Figure 1). *K. gracilis *leaf extract at the concentration of 50 *μ*g/mL potently inhibited 90% yield production of both viruses in RD cells (Figure 3). Also, IC_50_ value of *K. gracilis *leaf extract against EV71 and CVA16 were 35.88 *μ*g/mL and 42.91 *μ*g/mL, respectively (Figure 4), while the therapeutic index of *K. gracilis *leaf extract against EV71 and CVA16 ranged from 23 to 28. A literature survey indicated some extracts of Chinese herbs exhibiting anti-EV71 activities *in vitro*, such as *Houttuynia cordata* Thunb (IC_50_ = 125.9 *μ*g/mL) [38], *Woodfordia fruticosa* Kurz (IC_50_ = 1.2 *μ*g/mL) [34], *Salvia miltiorrhiza* (IC_50_ = 585 *μ*g/mL) [39], Pueraria lobata (IC_50_ = 0.028 *μ*g/mL) [40], and *Glycyrrhiza uralensis* (IC_50_ = 0.056 *μ*g/mL) [41]. Therefore, *K. gracilis *leaf extract exhibited moderately potent anti- EV71 and CVA16 activities.

Although pre- and post-treatments of* K. gracilis *leaf extract exhibited time differences, the two treatments reached 90% viral reductions (Figure 6), implying that antiviral activity of *K. gracilis *leaf extract could be associated with targeting viral proteolytic enzymes, viral RNA replication machinery, and host cell factors, and even reducing virus-induced cytopathogenesis, or inducing host antiviral responses. Because enterovirus 2A protease cleaves viral polyproteins and cellular factors in which regulates in apoptosis and nuclear-cytoplasmic trafficking of NF-*κ*B [31, 32], viral 2A protease was selected as the possible target by *K. gracilis* leaf extract. *In vitro* recombinant 2A protease and cell-based FRET assays indicated *K. gracilis *leaf extract inhibited the enzymatic activity of viral 2A proteases in a concentration-dependent manner (Figures 7 and 8). In addition, *K. gracilis* leaf extract significantly reduced the EV71-induced apoptosis, such as decreasing proform and active form of caspase 9 in EV71-infected cells (Supplemental Figure 3). Likewise, *K. gracilis *leaf extract abated the up-regulation of IL-6 and RANTES gene expressions due to EV71 and/or CVA16 infection (Figure 9) which correlated with down-regulation of virus-induced NF-*κ*B-mediated signaling (Supplemental Figure 2). Therefore, *K. gracilis* leaf extract exhibited an inhibitory effect on enteroviral 2A protease. This action might be in agreement with the decrease of virus-induced apoptosis and NF-*κ*B-mediated proinflammation. Our future perspective would be to purify and identify potent *K. gracili* compounds as viral 2A inhibitors.

It has been shown that significant increases in the levels of IL-1*β*, IL-6, IL-10, IL-13, IFN-*γ*, and TNF-*α* are detected in the serum and cerebral spinal fluid of EV71-infected patients [23, 42–45]. Moreover, elevation of IL-1*β*, IL-6, and TNF-*α* in CSF strongly correlates with clinical severity and is believed to be responsible for EV71-induced immunopathogenesis [26, 44, 46]. Clinically, treatment of anti-IL-6 neutralizing antibodies increases survival rates, reduces tissue damage, and activates immune cells, but could not affect the viral loads in EV71-infected neonate mice [47]. We demonstrated* K. gracilis *leaf extract concentration dependently reduced IL-6 mRNA levels upregulated by EV71 or CVA16 infection in RD cells (Figures 9(a)–9(c)). Moreover, we suggested significant inhibitory effects of *K. gracilis *leaf extract on* in vitro* replications of EV71 and CVA16 (Supplemental Figure 1, Figures 4–6) associated with its potent inhibition on viral 2A protease activities (Figures 7 and 8). Therefore, a combination of effective compounds of* K. gracilis *leaf extract shows a potential therapeutic approach against enteroviral infection.

In conclusion, *K. gracilis *leaf extract contains effective compounds with anti-enteroviral activities. *K. gracilis *leaf extract possesses potent effects in inhibiting enzymatic activities of viral 2A protease and reducing virus-induced expression of pro-inflammatory cytokines IL-6 and RANTES. *K. gracilis *leaf extract could be a safe and potential therapeutic agent against enteroviral infection.

## Supplementary Material

Supplemental Figure 1: Reduction of cytopathic effects by *K. gracilis* leaf extract. The morphology of RD cells infected with EV71 *(*A) or CVA16 *(*B) were observed for the effect of *K. gracilis* leaf extract in CPE reduction assay. EV71 and CVA16 at the MOI of 1 was each mixed with *K. gracilis* leaf extract, and then added into RD cell cultures. Incubated RD cells were observed and photographed under microscopy 24- and 48-h post infection.
Supplemental Figure 2: Phosphorylation of p38 MAPK, ERK1/2 and NF-**κ**B p65 in infected RD cells with or without the treatment of *K. gracilis* leaf extract. RD cells were infected with EV71 *(*A) or CVA16 *(*B) and simultaneously treated with *K. racilis* leaf extract at a concentration of 50 **μ**g/ml. The cells were harvested at 0-, 30-, 60- and 240 min post infection, and Western blotting analysis was performed as described in the Materials and Methods section.
Supplemental Figure 3: Western blotting of caspase 9 in EV71-infected RD cells with or without the treatment of *K. gracilis* leaf extract. RD cells were infected with EV71 at a MOI of 1 in the absence *(*Lane 1) and presence of *K. racilis* leaf extract at concentrations of 10, 50 and 100 **μ**g/ml *(*Lanes 2-4). The cells were harvested 1 day post infection, and Western blotting analysis was performed as described in the Materials and Methods section.
Supplemental Table 1: Virus loads in pooled intestines from EV71-inoculated suckling mice with or without treatment of *K. gracilis* leaf extract.
