# Supplementary material for: Antiviral Ability of Kalanchoe gracilis Leaf Extract against Enterovirus 71 and Coxsackievirus A16
Source: Evid Based Complement Alternat Med. 2012 May 15;2012:503165. doi: 10.1155/2012/503165 (PMC3361180; doi:10.1155/2012/503165)
Supplement: Supplementary file 1 — Supplemental Figure 1: Reduction of cytopathic effects by K. gracilis leaf extract. The morphology of RD cells infected with EV71 (A) or CVA16 (B) were observed for the effect of K. gracilis leaf extract in CPE reduction assay. EV71 and CVA16 at the MOI of 1 was each mixed with K. gracilis leaf extract, and then added into RD cell cultures. Incubated RD cells were observed and photographed under microscopy 24- and 48-h post infection. Supplemental Figure 2: Phosphorylation of p38 MAPK, ERK1/2 and NF-κB p65 in infected RD cells with or without the treatment of K. gracilis leaf extract. RD cells were infected with EV71 (A) or CVA16 (B) and simultaneously treated with K. racilis leaf extract at a concentration of 50 μg/ml. The cells were harvested at 0-, 30-, 60- and 240 min post infection, and Western blotting analysis was performed as described in the Materials and Methods section. Supplemental Figure 3: Western blotting of caspase 9 in EV71-infected RD cells with or without the treatment of K. gracilis leaf extract. RD cells were infected with EV71 at a MOI of 1 in the absence (Lane 1) and presence of K. racilis leaf extract at concentrations of 10, 50 and 100 μg/ml (Lanes 2-4). The cells were harvested 1 day post infection, and Western blotting analysis was performed as described in the Materials and Methods section. Supplemental Table 1: Virus loads in pooled intestines from EV71-inoculated suckling mice with or without treatment of K. gracilis leaf extract. [file 503165.f1.pdf]

Supplemental Fig.1.

A.

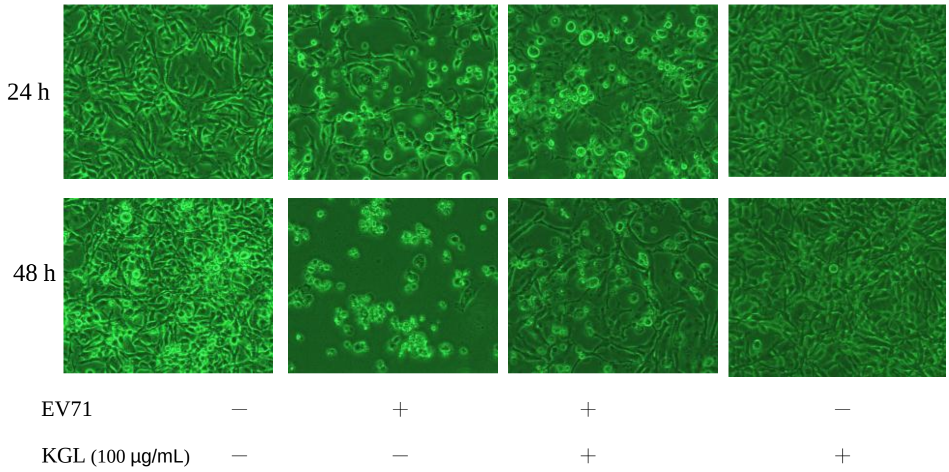

Supplemental Fig.1.

B.

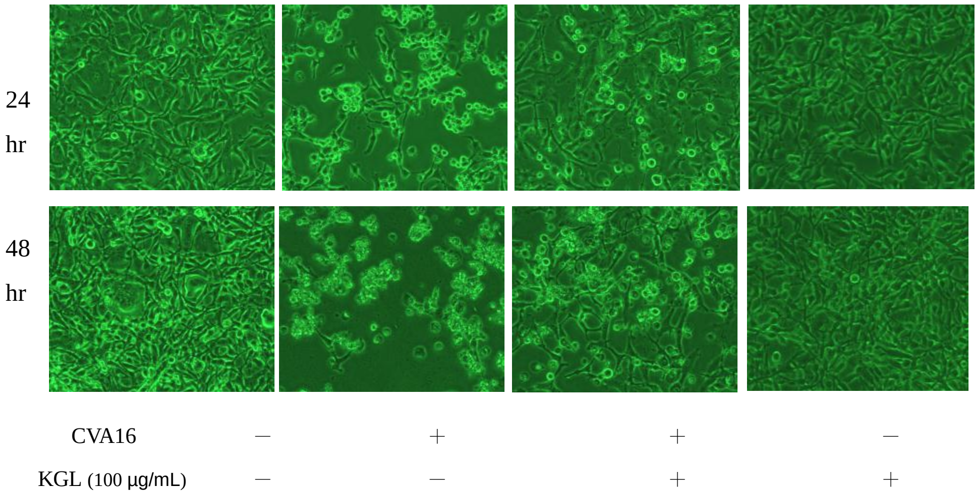

Supplemental Fig. 1. Reduction of cytopathic effects by *K. gracilis* leaf extract. The morphology of RD cells infected with EV71 (A) or CVA16 (B) were observed for the effect of *K. gracilis* leaf extract in CPE reduction assay. EV71 and CVA16 at the MOI of 1 was each mixed with *K. gracilis* leaf extract, and then added into RD cell cultures. Incubated RD cells were observed and photographed under microscopy 24- and 48-h post infection.

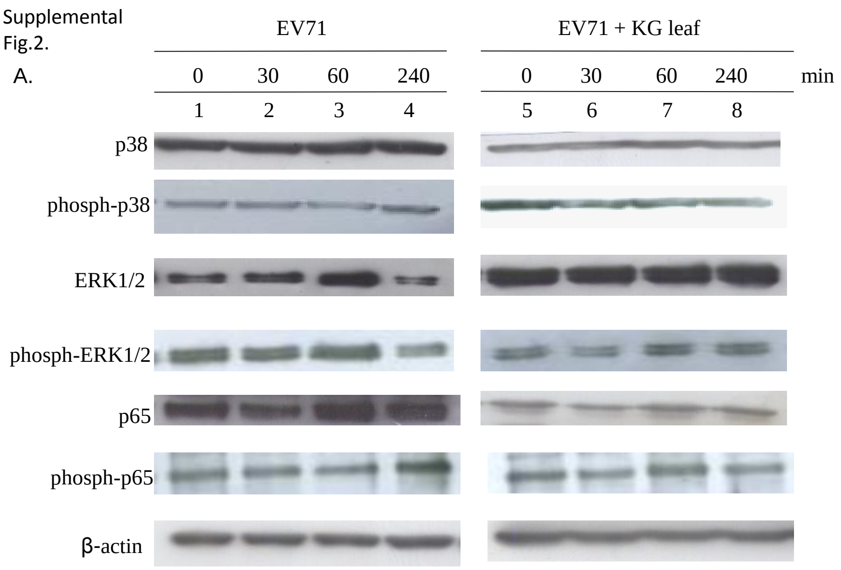

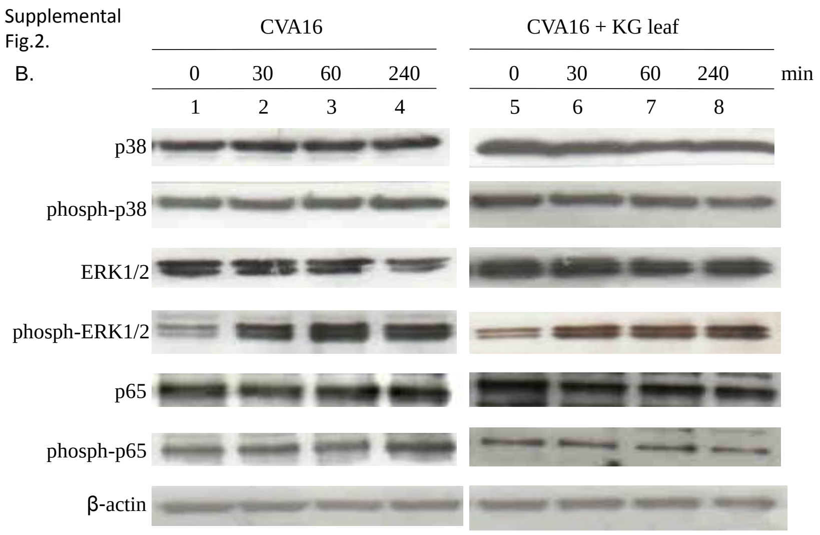

Supplemental Fig. 2 Phosphorylation of p38 MAPK, ERK1/2 and NF-κB p65 in infected RD cells with or without the treatment of *K. gracilis* leaf extract. RD cells were infected with EV71 (A) or CVA16 (B) and simultaneously treated with *K. racilis* leaf extract at a concentration of 50 μg/ml. The cells were harvested at 0-, 30-, 60- and 240 min post infection, and Western blotting analysis was performed as described in the Materials and Methods section.

Supplemental Fig. 3

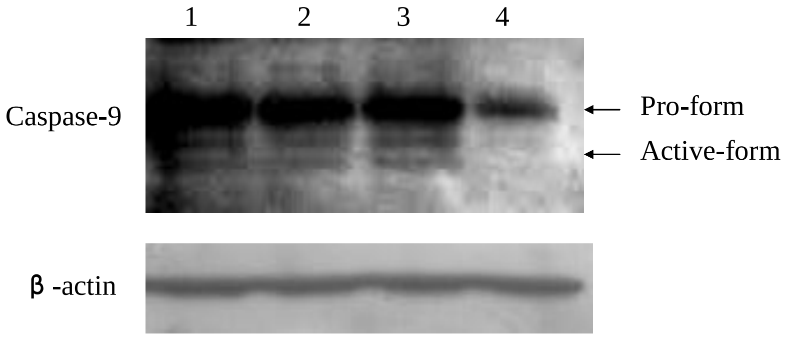

Supplemental Fig. 3. Western blotting of caspase 9 in EV71-infected RD cells with or without the treatment of *K. gracilis* leaf extract. RD cells were infected with EV71 at a MOI of 1 in the absence (Lane 1) and presence of *K. racilis* leaf extract at concentrations of 10, 50 and 100 μg/ml (Lanes 2-4). The cells were harvested 1 day post infection, and Western blotting analysis was performed as described in the Materials and Methods section.

Supplemental Table 1. Virus loads in pooled intestines from EV71-inoculated suckling mice with or without treatment of *K. gracilis* leaf extract

Group	Day 2 (pfu/ml)	Day 4 (pfu/ml)	Day 6 (pfu/ml)	Day 8 (pfu/ml)
0 mg/Kg KGL	3.7×10^{5}	3.1×10^{3}	2.8×10^{3}	ND
1 mg/Kg KGL	4.0×10^{5}	1.1×10^{3}	ND	ND
2 mg/Kg KGL	ND	ND	ND	ND

KGL: *K. gracilis* leaf extract; ND: not detectable
